# Utility of MRI for Characterizing Articular Cartilage Pathology in Dogs with Medial Coronoid Process Disease

**DOI:** 10.3389/fvets.2017.00025

**Published:** 2017-02-24

**Authors:** Samuel P. Franklin, Emily E. Burke, Shannon P. Holmes

**Affiliations:** ^1^Department of Small Animal Medicine and Surgery, Veterinary Teaching Hospital, University of Georgia, Athens, GA, USA; ^2^Regenerative Bioscience Center, University of Georgia, Athens, GA, USA; ^3^Veterinary Biosciences and Diagnostic Imaging, University of Georgia, Athens, GA, USA

**Keywords:** MRI, medial coronoid process, arthroscopy, dGEMRIC, elbow dysplasia

## Abstract

**Objective:**

To determine whether assessment of morphological MRI sequences or delayed gadolinium-enhanced MRI of cartilage (dGEMRIC) would have strong correlations with arthroscopic assessment of cartilage pathology in dogs with naturally occurring medial compartment pathology of the elbow.

**Methods:**

Dogs tentatively diagnosed with medial coronoid disease had evaluation of their affected elbows using radiography, morphological MRI sequences, and dGEMRIC MRI evaluation prior to arthroscopy. Elbow radiographs were graded 0–6 for severity of changes. Cartilage of the medial coronoid process (MCP) and humeral trochlea (HT) were scored on a 0–3 scale using anatomical MRI sequences. The T1 relaxation times for the MCP and trochlea were quantified using dGEMRIC. Cartilage pathology was graded arthroscopically using a modified Outerbridge score (MOS) by a surgeon blinded to MRI assessment. Correlations between radiography and MOS, and between MRI and MOS, were quantified.

**Results:**

Twenty-six elbows in 14 dogs were evaluated. There were statistically significant (*p* < 0.05) moderate correlations between radiographic scores and MOS for the MCP (*r* = 0.71) and HT (0.57). There was a statistically significant moderate correlation between morphological MRI scoring and MOS for the HT (*r* = 0.54; *p* < 0.05), but not for the MCP (*p* > 0.05). There was a weak, but significant correlation, between the dGEMRIC value and MOS of the MCP (*r* = 0.41; *p* < 0.05), but no correlation between the dGEMRIC values and MOS for the HT (*p* > 0.05).

**Clinical relevance:**

Statistically significant correlations to MOS were identified for both radiography and MRI but neither diagnostic modality provided sufficiently strong correlations to serve as a substitute for arthroscopic evaluation of the articular cartilage.

## Introduction

There is great variability in the character and severity of pathology affecting dogs with medial compartment disease of the elbow. Such variability includes varying degrees and distribution of cartilage damage on the medial coronoid process (MCP) or humeral trochlea (HT) with some dogs having arthroscopically normal or near normal articular cartilage in the medial compartment while other dogs have complete loss of articular cartilage throughout the medial compartment (1, 2). There is a need to accurately characterize pathology in the canine elbow non-invasively so that treatment planning can be performed prior to performing any surgery, including arthroscopic assessment. This is clinically relevant because approximately 50% of dogs with medial coronoid disease have some pathology of cartilage on the HT and there are a growing number of surgical options available for treating dogs when pathology is not limited solely to the MCP (3–7). An ability to accurately characterize pathology of the medial compartment non-invasively would facilitate selection of such treatments before diagnostic arthroscopy is performed. In addition, accurate non-invasive characterization of pathology would enable performance of clinical trials in which dogs managed non-surgically have pathology that is well defined. In turn, their response to non-surgical treatment could be compared to dogs managed surgically while being confident that the pathology affecting both groups were similar or equivalent.

Currently, radiography, computed tomography (CT), and arthroscopic surgery are the primary diagnostics used to make a diagnosis of elbow dysplasia (8). Of these, only arthroscopy provides an ability to assess the articular cartilage and is generally considered the gold standard for assessment of pathology in the canine elbow in dogs with MCP disease (2, 9, 10). Unfortunately, arthroscopy, although a relatively non-invasive treatment, is a relatively invasive diagnostic imaging assessment and staging tool. Conversely, MRI could potentially be used to non-invasively determine the pathology in the canine elbow. A few studies have evaluated the use of MRI for such purpose (11–14). Initial work demonstrated good sensitivity and specificity of MRI for diagnosing pathology of the MCP and HT in comparison to radiography while using surgical findings as the gold standard (11). However, there were limitations of the technique and so use of MRI arthrography was subsequently evaluated but failed to identify suspected clinically relevant information in some cases (12).

A more recent investigation evaluated standard morphological MRI sequences (using a 1.5-T scanner) and their correlation to arthroscopic and histopathologic assessments in dogs with subtle radiographic changes and only those having arthroscopically normal HT. There was a statistically significant but only moderate correlation between the MRI score and the modified Outerbridge score (MOS) (14). The authors concluded that the results were inadequate to validate use of MRI as a stand-alone staging tool. Another study evaluated the repeatability of values obtained using quantitative MRI techniques, delayed gadolinium-enhanced MRI for cartilage [delayed gadolinium enhanced MRI of cartilage (dGEMRIC)], and T2 mapping specifically, in six research dogs with normal elbows (15). Both intra- and interobserver precisions were found to be good. These results are encouraging and raise the possibility that MRI could potentially be used to identify pathology of the articular cartilage early in the course of disease and could also potentially monitor response to treatments. However, no study has yet to demonstrate a sufficiently strong correlation between MRI scoring and either arthroscopy or biochemical analysis of the articular cartilage in dogs with naturally occurring pathology of the medial compartment to justify routine clinical application.

The purpose of this study was to determine whether morphological or quantitative MRI assessment of articular cartilage pathology correlates with arthroscopic assessments in dogs with naturally occurring pathology of the medial compartment. Our second objective was to determine whether radiographic assessment of pathology correlated with arthroscopic assessed severity of joint pathology as has been previously demonstrated (1). We hypothesized that correlations between MRI and arthroscopic scoring and between radiographic and arthroscopic scoring would both be statistically significant. We hypothesized that the strength of correlation would be greater between MRI and arthroscopy than between radiography and arthroscopy.

## Animals and Methods

This study was approved by the Clinical Research Committee at the University of Georgia.

### Animals

Dogs that were tentatively diagnosed with naturally occurring medial coronoid disease based upon history, physical examination, and radiography and that were candidates for general anesthesia and arthroscopy were recruited for enrollment. Owners were provided with a written description of the study and owners provided written consent for inclusion of their dog in the study. These dogs would not have had MRI at our institution for suspected medial coronoid disease if they were not enrolled in this research study. The owner did not pay for the MRI as funding for the MRI was provided by the study sponsor.

### Radiography

If dogs had recent (within 6 weeks), well-positioned, adequate quality medial–lateral and cranial–caudal radiographs of the affected elbows prior to presentation to the UGA VMC, radiography was not repeated. If appropriate radiographs were not available at the time of presentation, dogs were sedated with 0.005 mg/kg of Dexmedetomidine (Pfizer, NY, USA) and 0.5 mg/kg Nalbuphine (Pfizer, NY, USA) given intravenously and orthogonal radiographs of the affected elbows were made.

### MRI Protocol

Dogs were anesthetized for MRI evaluation with routine anesthetic protocols that typically included premedication with an opioid and a sedative followed by induction with Propofol (PropoFlo 28, Zoetis, Kalamazoo, MI, USA) or Ketamine (Ketaset, Zoetis, Kalamazoo, MI, USA) and maintained with Isoflurane or Sevoflurane dependent upon anesthesiologist choice. The MRI examinations were performed using a 3.0-T MRI unit (Skyra, Siemens). Since bilateral studies were performed, the dogs were positioned in sternal recumbency with their thoracic limbs extended forward. Care was taken to ensure straight alignment of the joints along the long axis of the thoracic limbs. Parallel imaging was employed with a combination of a phased-array spine coil and a flexible body matrix coil, respectively positioned ventral and dorsal to the elbows. The imaging protocol started with the morphologic sequences, including intermediate-weighted (IW) fat-suppressed fast spine echo (FSE) (sagittal plane), IW fat-suppressed FSE (dorsal plane), proton density (PD) FSE (sagittal plane), and PD SPACE (sagittal plane) imaging. The dGEMRIC series were collected following the morphologic series and T1 MapIT sequence in the sagittal plane. A pre-contrast series was collected of both elbows after which the dogs were administered 0.01 mmol/kg of gadopentetate dimeglumine (Magnevista^®^, Bayer, Pittsburgh, PA, USA) intravenously. The elbows were cycled through a range of motion (flexion, extension, and rotational motion) for 10 min while the dog was still anesthetized, similar to a previously described protocol (15). If both elbows were to be evaluated, they were cycled through range of motion simultaneously. Following range of motion, the dogs were repositioned in sternal recumbency with the limbs pulled forward and the post-contrast dGEMRIC images were collected.

### Arthroscopic Assessment and Treatment

Immediately following completion of MRI and under the same general anesthesia, dogs were aseptically prepared for surgery. Arthroscopy was performed by a surgeon blinded to MRI findings using a standard caudomedial instrument portal and a craniomedial instrument portal and using 1.9 mm 30° foreoblique arthroscope (Arthrex Vet Systems, Naples, FL, USA). The specific structures evaluated included the synovium and articular surface of the radial head, humeral capitulum, HT, MCP, lateral coronoid process, and the semilunar notch. The arthroscopic procedure was recorded. Treatment of the affected MCP included a combination of fragment removal and/or subtotal coronoid ostectomy depending upon the extent of pathology. All dogs were recovered from general anesthesia and postoperative pain control provided with intermittent opioid administration for 24 h and approximately 14 days of non-steroidal anti-inflammatory medication.

### Arthroscopic Scoring

The recorded videos were reviewed by the surgeon who was blinded to MRI findings for characterization of pathology. Pathology of the MCP and the HT was scored using a MOS (0–5) that has been described and used in prior studies for characterization of elbow pathology in dogs with medial compartment pathology (Table 1) (1, 3).

**Table 1:** **Modified Outerbridge scoring system used for arthroscopic evaluation of cartilage pathology (1, 3)**.

Modified Outerbridge score	Description of gross cartilage quality
0	Normal
1	Chondromalacia, determined in part by probing with an arthroscopic probe
2	Partial thickness fibrillation
3	Deep fibrillation
4	Full thickness cartilage loss
5	Subchondral bone eburnation

### Radiographic Scoring

All radiographs had all identifying information removed, were randomized, and assigned a novel identification number to create blinded assessment. Radiographs were assessed by the surgeon a minimum of 6 months following the last arthroscopy. The medial–lateral radiographs were scored on a 4-point (0–3) scale using a previously described system (1). Cranial–caudal radiographs were also scored on a 4-point scale in which osteophyte formation was considered with a score of 0 representing a normal elbow and 1, 2, and 3 were consistent with mild, moderate, and severe radiographic changes, respectively. Scores for both images were summed to provide a maximum score of 6, representing the most substantial radiographic changes.

### MRI Scoring

The morphologic MRI images were graded by a radiologist blinded to arthroscopic and radiographic findings. Cartilage of the MCP and HT were each scored on a 0–3 scale (0, no change in signal from the cartilage; 1, signal change, but no change in thickness of the associated articular cartilage; 2, signal change and partial thinning of the cartilage; 3, full thickness cartilage loss).

**Figure 1:**
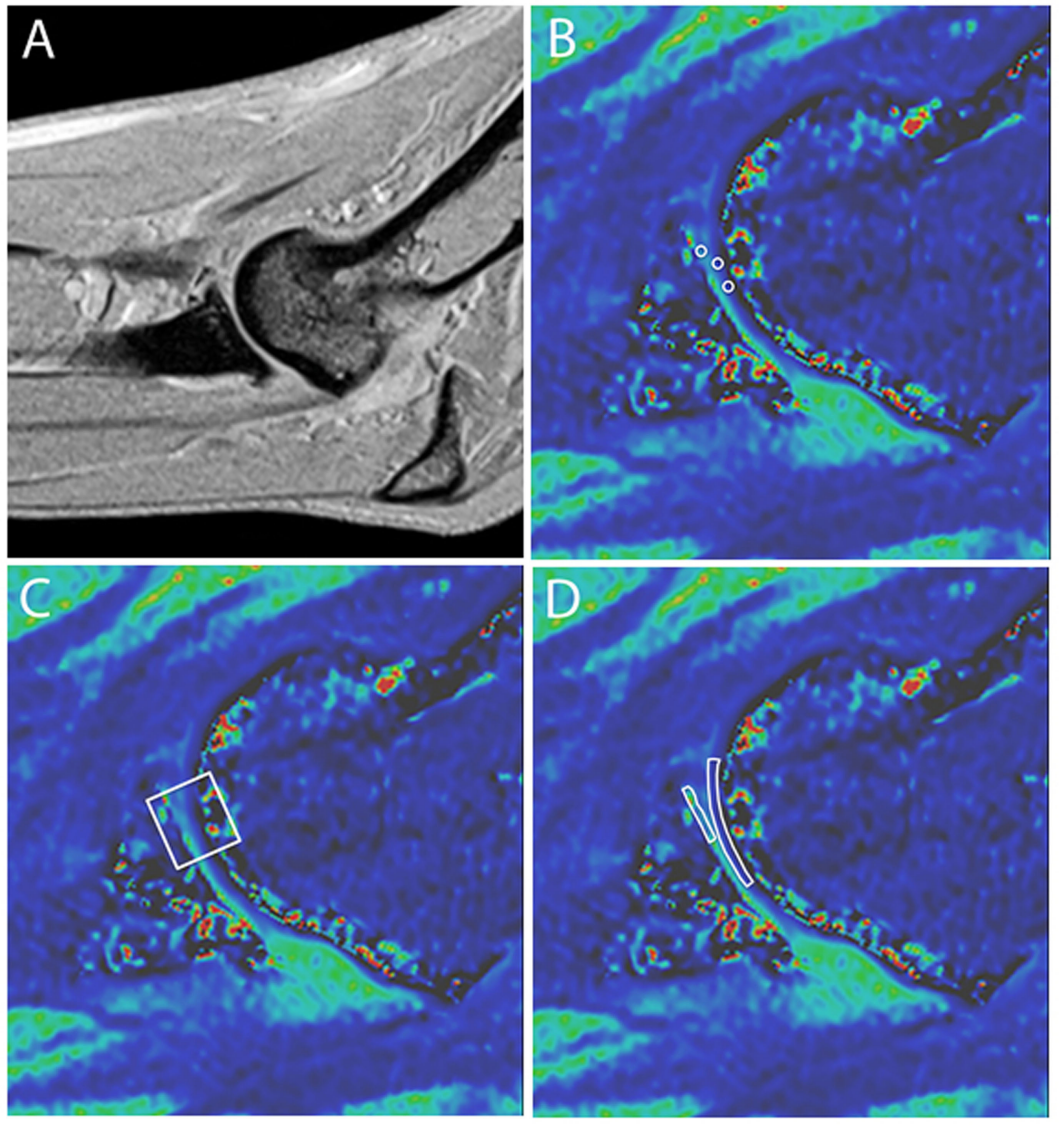
**Representative sagittal plane MRI images**. **(A)** T1-weighted anatomical reference image and **(B–D)** magnified delayed gadolinium-enhanced MRI of cartilage images depicting three methods for drawing regions of interest (ROIs) for cartilage scoring. **(B)** One ROI is on the medial coronoid process (MCP) and two on the humeral trochlea (HT). Note that the ROIs include the articular cartilage layer without substantial inclusion of adjacent subchondral bone, joint fluid, or opposing articular surface. **(C)** A single square ROI is used that spans the joint space and includes the articular cartilage of the medial HT and MCP. **(D)** Two free-form ROIs are used that trace the MCP and HT.

Evaluation of the dGEMRIC images was performed by the same radiologist blinded to arthroscopic and radiographic findings. The radiologist evaluated the dGEMRIC images a minimum of 3 months following evaluation of the morphologic MRI images and did not review the morphologic images while evaluating the dGEMRIC images so as to obviate any influence on the dGEMRIC evaluations by prior assessment of the morphologic images. Three consecutive sagittal plane slices were assessed that included the MCP and HT. On each of these slices, three different methods of creating regions of interest (ROIs) were performed. The first method included creating one small ROI on the MCP and two small ROIs of the same dimension on the weight-bearing articular surface of the HT (Figures 1A,B). Each of these ROIs was created and placed to include the articular cartilage without including underlying subchondral bone or overlying joint fluid. Accordingly, three and six quantitative measurements of the T1 relaxation time were obtained for the MCP and HT, respectively. The means of these were calculated and used in subsequent statistical analyses. Small circular ROIs were used to ensure that the area encircled with each anatomic region was constant and to ensure that none of these ROIs spanned the joint space to include the opposing articular surface. In addition, use of small ROIs enabled placement to include the articular cartilage while excluding measurement from the adjacent synovial fluid or subchondral bone.

In an effort to more closely replicate the previous study documenting consistency in ROI measurements within and between observers using dGMERIC in the canine elbow, measurements were also taken by placing a single larger square ROI that spanned the joint space and incorporated the cartilage surface of the HT, any intervening joint fluid, and the articular surface of the MCPs (Figure 1C) (15). Such measurements were made on the same three sagittal images and the mean calculated for subsequent statistical analysis. For the third method of creating ROIs, free-form traces of the MCP and the HT were performed (Figure 1D). Measurements were made on the same three sagittal images and the means calculated.

### Statistical Analysis

All correlations were assessed using Pearson product-moment correlations. Specifically, the correlation between the MOS for the MCP and the radiographic score and between the MOS for the HT and radiographic score were quantified. Similarly, correlations between the arthroscopic MOS for the MCP and the MRI score for the MCP using the morphologic images was quantified. Likewise, the correlation between the arthroscopic MOS for the HT and the MRI score for the HT using the morphologic images was determined. Correlations between the MOS and the dGEMRIC scores were similarly calculated using the dGEMRIC values obtained from each of the three different techniques of drawing ROIs (Table 2).

**Table 2:** **Correlations of diagnostic evaluations to arthroscopic assessment of cartilage pathology**.

Diagnostic imaging modality	Anatomic location	MOS location	*r*	*p*-Value
Radiography	Global elbow score	MCP	0.71	<0.0001
Radiography	Global elbow score	Trochlea	0.57	<0.01
MRI	MCP (morphologic images)	MCP	0.22	0.28
MRI	Trochlea (morphologic images)	Trochlea	0.54	<0.01
Delayed gadolinium enhanced MRI of cartilage (dGEMRIC)	MCP circular region of interest (ROI)	MCP	0.25	0.23
dGEMRIC	Trochlea circular ROIs	Trochlea	−0.14	0.50
dGEMRIC	Square ROI spanning MCP/human trochlea (HT)	MCP	0.41	0.04
dGEMRIC	Square ROI spanning MCP/HT	Trochlea	0.10	0.65
dGEMRIC	MCP free-form trace	MCP	0.35	0.09
dGEMRIC	Trochlea free-form trace	Trochlea	−0.06	0.76

*MOS, modified Outerbridge score; MCP, medial coronoid process*.

*Trochlea refers to the HT*.

## Results

### Demographics

Radiography, MRI, and arthroscopic evaluations were performed in 26 elbows in 14 dogs; 2 dogs had only one elbow evaluated. There were 2 females, 6 spayed females, 4 castrated males, and 2 intact males included. Breeds included Labrador Retriever (*n* = 5), Golden Retriever (*n* = 2), mixed breed (3), and one each of Anatolian Shepherd, Newfoundland, Pit Bull, and Rottweiler. The mean age was 3.1 years (±SD 4.0 years). The mean body weight was 35.0 kg (±10.1). T1 relaxation times using dGEMRIC were assessed from 25 elbows in 14 dogs because the associated sequence was lost prior to analysis for one elbow of 1 dog.

### Diagnostic Imaging Assessment

#### Radiographic and MRI Assessment

The mean radiographic score for each elbow was 3.0 (±1.8) out of 6. The mean grade for articular cartilage on the MCP using MRI anatomical sequences was 2.2 (±0.6). The mean MRI score for the humeral trochlear cartilage using MRI anatomical sequences was 1.68 (±0.8).

#### Delayed Gadolinium-Enhanced MRI of Cartilage

The mean time between gadolinium administration and associated imaging was 37 min (±13). If only those elbows imaged first following contrast administration were considered the mean time between contrast administration and the beginning of image acquisition was 29 min (±8) and the mean delay between contrast administration and beginning of image acquisition for the second elbow was 48 min (±12). The mean T1 relaxation times for the assessed areas are presented in Table 3.

**Table 3:** **Mean delayed gadolinium enhanced MRI of cartilage T1 relaxation times**.

Anatomic location	Region of interest shape	Mean (±SD) T1 relaxation time
MCP	Small circular	728.1 (±228.5)
Trochlea	Small circular (#1)	723.2 (±274.8)
Trochlea	Small circular (#2)	734.0 (±267.5)
MCP and trochlea	Square	647.5 (±159.7)
MCP	Free-form trace	808.5 (±337.4)
Trochlea	Free-form trace	788.2 (±258.5)

*MCP, medial coronoid process*.

*Trochlea (humeral trochlea)*.

#### Arthroscopic Assessment

The mean MOS for cartilage pathology on the MCP was 1.8 (±1.4). The mean MOS for cartilage pathology on the HT was 1.5 (±1.6).

### Correlations

**Figure 2:**
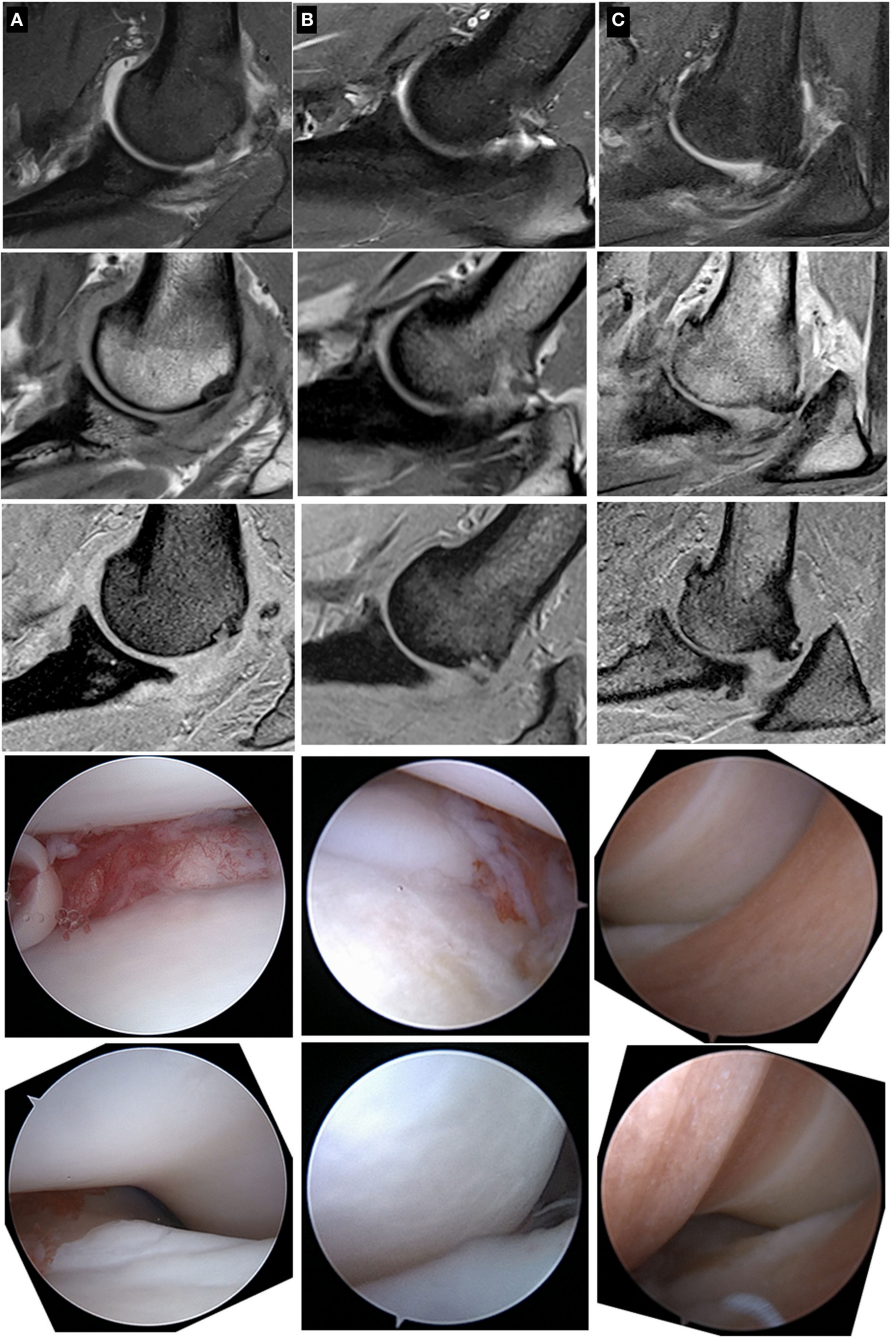
**Sagittal plane MRI images and associated arthroscopic images from three patients (patients A, B, and C) with cartilage pathology of varying severity**. From top to bottom within a column, the images are fat-suppressed proton density (PD)-weighted (2.5 mm thick), PD-weighted (1.5 mm thick), and T1-weigthed (1.0 mm thick) images followed by a representative arthroscopic image of the medial coronoid process and the humeral trochlea. Note that the spectrum of cartilage pathology is readily apparent with the arthroscopic images, but less apparent with the MRI images.

There were numerous statistically significant correlations between the arthroscopic MOS and diagnostic imaging (Table 2). The strength of correlations between radiographic scoring and arthroscopic MOS were moderate. There was moderately strong (*r* = 0.54) statistically significant (*p* < 0.05) correlation between MRI cartilage assessment of the HT and MOS using morphological sequences; there was not a significant correlation between MRI cartilage assessment of the MCP and MOS of the MCP. Three out of five MCPs that had full thickness cartilage loss (MOS Grade IV or V) were assessed as having only partial thickness cartilage loss using the morphological MRI sequences (see Figure 2). Two out of three HTs that had MOS Grade IV or V change were assessed as only having partial thickness cartilage loss using the morphological MRI sequences (Figure 2). There was a weak to moderate (*r* = 0.41) statistically significant (*p* < 0.05) correlation between the T1 relaxation time using the dGEMRIC sequences and MOS for the MCP when using a single square ROI that spanned the joint space and included the articular surface of the MCP and HT. There were no other statistically significant correlations between the MOS and T1 relaxation times using dGEMRIC sequences (Table 2).

## Discussion

The demographic data demonstrate inclusion of dogs of typical breeds (large breeds) affected with elbow pathology but with a wide range in age and degree of arthroscopic assessed cartilage pathology. The mean MOS score for the MCP and HT were relatively low at 1.8 and 1.5, respectively. Although these values are somewhat low and might be interpreted as hampering our ability to detect significant correlations between the MOS and MRI scores that SDs of these values were relatively high and the MOS values ranged from 0 to 5 for both the MCP and HT. The inclusion of dogs with notable variability in severity of cartilage pathology was specifically desired to maximize the likelihood of detecting positive correlations between non-invasive diagnostic imaging modalities and the arthroscopic assessed cartilage pathology. Indeed, these data do demonstrate statistically significant but only moderate correlations between radiographic assessment and arthroscopic assessments of cartilage pathology in the medial compartment of the canine elbow. These results warrant accepting our first hypothesis and are not surprising given results from a previous study demonstrating a relationship between the arthroscopic global cartilage pathology score and radiographic assessment of disease severity in a large group of Labrador Retrievers (1). The results from that study enables one to make probability statements regarding severity of cartilage pathology based upon radiographs but do not enable accurate prediction of cartilage pathology in many dogs with mild or moderate (radiographic score of 1 or 2) radiographic changes (1). The data from this study are based upon far fewer dogs and do not provide evidence for greater clinical utility of radiographic examination than that provided in the previous study (1). As a result, we conclude that there is a moderate correlation between radiographic findings and arthroscopically assessed cartilage pathology but there remains a notable possibility that dogs with minimal or moderate radiographic change have severe cartilage pathology as assessed arthroscopically (16). As a result, radiographs remain insufficiently sensitive for identification of all dogs with end stage medial compartment disease. In turn, radiographs in isolation are inadequate for staging or outcome assessment in clinical trials comparing non-surgical management to surgical treatment for dogs with pathology of the medial compartment.

There was also a statistically significant correlation between MOS for the HT and assessment of cartilage pathology using the MRI morphological images. However, this correlation was weak to moderate and judged to be clinically irrelevant and there was no correlation between MRI assessment using the morphological images and the MOS for the MCP. As a result, we do not accept our hypothesis that correlations between MRI assessment (using morphological images) and arthroscopy would be stronger than the correlations between radiographic scoring and arthroscopic assessments of cartilage pathology. These results, although disappointing, are not unprecedented as a recent study with similar objectives also failed to find highly clinically relevant correlations between MRI assessment of canine elbows using morphological images and arthroscopic assessment of the elbows (14). The data from these two studies demonstrate that the morphpological MRI protocols used in these studies cannot be reliably used to fully stage elbow pathology. This conclusion is made with consideration to the fact that the signal to noise ratio, high bandwidth, and image resolution were maximized in this 3.0-T MRI study using longer scan times than typical for routine clinical scans. Specifically, image resolution and bandwidth were used to have multiple pixels within the 0.4–0.6 mm thick cartilage layer to facilitate identification of partial and full thickness cartilage loss (17). Thinner slices were also obtained but partial volume averaging effects were noted, particularly on the HT, which potentially could mask lesions with the mild curvature of the structure in the sagittal plane. We do not believe that poor quality images can be an explanation for failing to more accurately identify cartilage pathology with MRI. Possible explanations for inaccuracy, or rather opportunities for re-evaluation and improvement of MRI for cartilage assessment in the canine elbow may include using different sequences (i.e., 3D submillimeter isotropic FSE images) and imaging in different planes than what was used in this study. The sagittal plane was selected to obtain high in-plane resolution of cartilage contours perpendicular to the orientation of the majority of the cartilage line the MCP and HT. Based on this study, the MRI reviewer’s impression was that at most one morphologic slice was truly perpendicular to the articular surface and would not suffer partial volume effects due to the small anatomy and the bidirectional curvature of the MCP and HT.

There was also a moderate and statistically significant correlation between the dGEMRIC T1 relaxation time and MOS for the MCP using a single ROI that spanned the articular surface. This result is interesting in that this was the only method of drawing ROIs, of those assessed in this study, that demonstrated a significant correlation and most closely resembles the ROI methodology used in previous study demonstrating repeatability of measurements in dogs with normal elbows (15). For those reasons, this significant finding is encouraging that this methodology could ultimately become clinically useful with further validation. However, the correlation identified in this study is considered too weak to be clinically applicable. As a result, we are unaware of any studies demonstrating a clinically relevant correlation between dGEMRIC values and arthroscopic assessment in canine elbows with naturally occurring disease.

There are several possible explanations as to why the dGEMRIC values did not correlate more strongly with the arthroscopic assessed images in these elbows. First, it is possible that the arthroscopic assessment does not correlate well with the biochemical composition and histology of the cartilage and that the MRI values may have correlated more closely with biochemical asssessments of the cartilage.

A recent study demonstrated only weak correlations between histology and MRI interpretations from anatomical sequences (14). Second, it is possible that the protocol for gadolinium administration, passive range of motion, and relatively short delay until image acquisition did not result in sufficient gadolinium penetration into the joint and affected cartilage. When considering that the time frame between gadolinium administration and image acquisition is shorter in this study than in a previous study evaluating dGEMRIC for normal canine elbows, this seems a possible explanation (15). However, comparison of the dGEMRIC values for the HT and humeral capitulum from this study (data not shown) demonstrated significantly higher T1 relaxation rates in the lateral compartment, consistent with at least some gadolinium entering the joint and decreasing the T1 relaxation times in the medial compartment in comparison to the lateral compartment. Last and with relevance to use of morphological images as well as quantitative MRI, the cartilage in the canine elbow is quite thin with mean cartilage thickness of just 0.51 mm in one study (17). Assessment of cartilage status in this anatomic location using either morphological images or quantitative MRI may always be hampered by the thinness of the cartilage.

Conclusions that can be drawn from negative results are tempered by associated study limitations. Further limitations of the study beyond those mentioned above include that intra- and interobserver repeated measures were not quantified for the radiographic, arthroscopic, or MRI assessments. However, demonstration of precision is more pertinent when positive results are obtained, and it is necessary to show that such results can be repeated. The greatest limitation is that biochemistry and histologic assessment of tissue samples was not possible in this study using client-owned dogs and it remains feasible that MRI may have correlated more closely with histology or biochemistry than with the arthroscopic evaluations. No tissue was removed from the HT and the subtotal coronoidecotmies performed in this study were typically performed with an arthroscopic burr to mitigate the risk of creating intra-articular free bodies as has been documented following arthroscopic treatment of medial coronoid disease (18). Large subtotal coronoidectomies were not performed based upon some concern that such approach may not provide an optimal outcome (19). As a result, larger portions of bone and cartilage were not available for biochemical or histological evaluation.

Despite these limitations, we conclude that the correlations between arthroscopic findings and interpretations of MRI images were absent to moderate. Importantly, the morphological MRI images evaluated in this study did not consistently enable identification of elbows with full thickness cartilage loss on the HT (Figure 2). As a result, MRI cannot be substituted for arthroscopic assessment at this point in time, a conclusion also obtained by other investigators using morphological MRI assessments as well as using CT evaluations of canine elbows (10, 14).

## Author Contributions

SF devised the study, performed surgery, evaluated data, and prepared the manuscript. EB performed MRI evaluations. SH developed the MRI protocols, evaluated the MRI images, and contributed to manuscript preparation.

## Conflict of Interest Statement

The authors declare that the research was conducted in the absence of any commercial or financial relationships that could be construed as a potential conflict of interest.
